# Lithium-induced cognitive dysfunction assessed over 1-year hospitalisation: A case report

**DOI:** 10.4102/sajpsychiatry.v30i0.2314

**Published:** 2024-09-26

**Authors:** Yuji Murase, Masaki Kato, Toshihiko Kinoshita, Yoshiteru Takekita

**Affiliations:** 1 Department of Neuropsychiatry, Faculty of Medicine, Kansai Medical University, Osaka, Japan

**Keywords:** SILENT, lithium-induced neurotoxicity, irreversible neurological sequelae, overdose, cognitive dysfunction, neuropsychological tests, COGNISTAT, WAIS

## Abstract

**Introduction:**

Lithium-induced neurotoxicity is almost always reversible but can cause irreversible neurological sequelae, namely the syndrome of irreversible lithium-effectuated neurotoxicity (SILENT). As there is no definitive treatment for SILENT, caution is required when administering lithium. Reports on the effect of lithium-effectuated neurotoxicity on cognitive function are limited. We report a case in which high cognitive function was lost after lithium overdose and hardly recovered, as evaluated using multiple neuropsychological tests during a 1-year hospitalisation period.

**Patient presentation:**

A 52-year-old man on lithium medication with bipolar disorder was admitted to the intensive care unit because of lithium overdose. The patient achieved lucid consciousness after continuous haemodiafiltration. However, he could not move his body as desired or produce appropriate verbal expressions; thus, he was moved to our psychiatric ward, where his treatment continued.

**Management and outcome:**

After several months, the patient was diagnosed with SILENT owing to persistent motor and cognitive dysfunctions. Multiple neuropsychological tests were performed, and cognitive function was evaluated. The Neurobehavioural Cognitive Status Examination showed a worsening trend, and the full intelligence quotient of the Wechsler Adult Intelligence Scale-Third Edition was in the mild intellectual disability range.

**Conclusion:**

This is a clear case of cognitive dysfunction due to SILENT and is difficult to treat. Thus, it is crucial to prevent the onset of SILENT.

**Contribution:**

This report is valuable because it is one of the few to track changes in cognitive function over time in a patient with SILENT using objective measures over 1 year of hospitalisation.

## Introduction

Lithium is widely used to treat bipolar disorder; however, its administration should be carefully monitored. Schneider et al.^1^ reported that lithium administration causes side effects, including lithium-induced neurotoxicity, even at therapeutic levels. Kores et al.^2^ reported that neurotoxicity is almost always reversible but can lead to the syndrome of irreversible lithium-effectuated neurotoxicity (SILENT). According to Adityanjee and Schou,^3,4,5^ SILENT is a condition in which neurological dysfunction caused by lithium persists 2 months after discontinuation. Symptoms include persistent cerebellar dysfunction, extrapyramidal symptoms, brainstem dysfunction and cognitive deficits. Additionally, sequelae usually persist at the 1-year follow-up.^6^ While there is no definitive treatment for SILENT,^7^ the main treatment is physical rehabilitation.^6^ Most reports on SILENT have focused on motor impairment, with few detailing cognitive decline.^8^ Here, we report a patient in whom cognitive function decreased after lithium overdose and hardly recovered, as evaluated using multiple neuropsychological tests during a 1-year hospitalisation period.

### Ethical considerations

Written consent was obtained from the patient for the presentation of this case.

### Patient presentation

A 52-year-old man who had been admitted to another hospital for a lithium overdose was transferred to our hospital because the first hospital did not have a psychiatric ward. The patient was diagnosed with bipolar disorder at 40 years of age, and lithium treatment was initiated. After graduating from high school, he started working for a major railroad company and progressed to the position of assistant station master. However, he was divorced and demoted from his job due to domestic violence related to his drinking. At that time, he had a diagnosis of alcohol use disorder but had no history of continuous or heavy drinking.

He was brought to the emergency department because of lithium overdose, after which he was admitted to the intensive care unit because of unconsciousness, with a Glasgow Coma Scale (GCS) score of 3 (E1V1M1). Blood tests revealed elevated lithium (3.79 mEq/L) and creatinine (7.14 mg/dL) levels, which led to a diagnosis of lithium toxicity. The overdose accounted for 9.6 g of lithium, as calculated from the large number of empty packets in his possession. After continuous haemodiafiltration for 40 h and intravenous rehydration, he achieved lucid consciousness with a GCS score of 15 (E4V5M6), and his lithium and creatinine levels decreased (1.02 mEq/L and 1.02 mg/dL, respectively).

The patient was transferred to the psychiatric ward of our hospital on day 35 of hospitalisation. He exhibited truncal ataxia, dysarthria, intentional tremors, coordinated movement disorders such as loss of manual dexterity and unremarkable blood test results. Blood lithium carbonate levels were below measurement sensitivity thresholds. Magnetic resonance imaging of the head showed chronic ischaemic changes. Cerebrospinal fluid examination and electroencephalography revealed no abnormalities. He often became frustrated with his word-finding difficulty and coordination difficulty, or exploded in anger over trivial things, but each time these subsided spontaneously within a few hours to a day or so.

## Management and outcome

**FIGURE 1:**
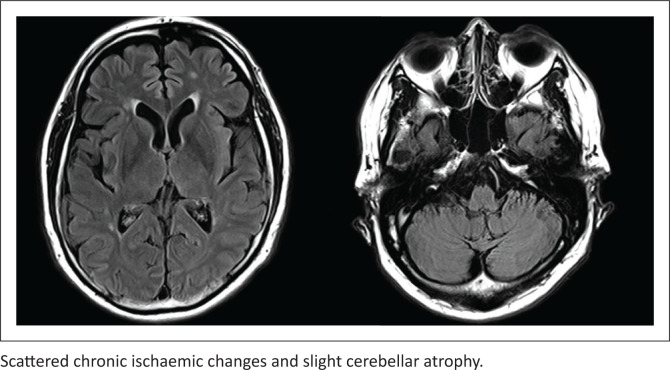
Axial Fluid-Attenuated Inversion Recovery (FLAIR) images of the brain on day 242.

Severe cerebellar ataxia and dysarthria persisted several months after transfer to our hospital; therefore, the patient was diagnosed with SILENT. Rehabilitation was provided for persistent functional impairment, during which cognitive function was assessed in detail using the Japanese version of the Neurobehavioural Cognitive Status Examination (COGNISTAT) and Wechsler Adult Intelligence Scale-Third Edition (WAIS-III). Neurobehavioural Cognitive Status Examination, performed twice, showed a worsening trend, with a particularly noticeable decrease in standard scores for orientation and construction. The full intelligence quotient of WAIS-III was in the mild intellectual disability range (Table 1). Magnetic resonance imaging of the head performed on day 242 showed cerebellar atrophy compared to that on admission (Figure 1). The diagnosis was eventually changed from bipolar disorder to organic mental disorder due to SILENT, and he was followed up without drug treatment. Even then, no mood symptoms were observed. Motor and cognitive functions hardly improved, and he was unable to resume employment after discharge on day 390.

**TABLE 1:** Results of neuropsychological tests.

Test	Evaluation	Subscale or factor	Day	Score	Day	Score
COGNISTAT	Standard score†	Orientation	23	8	226	5
		Attention	-	10	-	10
		Comprehension	-	10	-	10
		Repetition	-	9	-	9
		Naming	-	10	-	10
		Construction	-	7	-	7
		Memory	-	9	-	10
		Calculation	-	10	-	8
		Similarities	-	10	-	10
		Judgement	-	9	-	8
WAIS-III	IQ	Full IQ	43	63	-	-
		Verbal IQ	-	73	-	-
		Performance IQ	-	58	-	-
	Group index	Verbal comprehension	-	90	-	-
		Perceptual organisation	-	63	-	-
		Working memory	-	67	-	-
		Processing speed	-	52	-	-

COGNISTAT, the Neurobehavioural Cognitive Status Examination; WAIS-III, Wechsler Adult Intelligence Scale-Third Edition; IQ, Intelligence Quotient.

† , 9 or higher = normal; 8 = mild disability; 7 = moderate disability; 6 or lower = severe disability.

## Discussion

The patient had worked for a well-known railroad company and had been repeatedly promoted, suggesting that his cognitive function was originally high, although there were no objective measures, and declined after the lithium overdose. Based on the COGNISTAT results, apart from worsening disorientation, which seemed to be influenced by prolonged hospitalisation, constructional disability, which interfered with his daily life, was particularly noticeable. Additionally, the WAIS-III results showed that verbal comprehension remained in the average range, but processing speed, working memory and perceptual organisation were low. The results suggest a decline beyond what would be expected due to physical disability such as reduced dexterity, indicating that there would be persistent visual cognitive and memory impairments, executive dysfunction and attention impairment, in particular. These cognitive impairments are similar to those found in the former report,^8^ which lends credibility to this report. Despite continuous rehabilitation, his cognitive function hardly improved after 1 year and even worsened in some aspects. Although the neurological damage mechanism in SILENT is unclear, magnetic resonance imaging of the head shows cerebellar or cerebral atrophy.^6,9,10,11^ To our knowledge, no other imaging findings have been reported yet. Here, the only characteristic finding, other than ischaemic changes, was cerebellar atrophy. As it is challenging to treat SILENT, its incidence must be avoided in patients using lithium. This report is one of the few to monitor gradual changes in cognitive function immediately after SILENT onset to 1 year during hospitalisation using objective measures.
